# Axillary silicone lymphadenopathy presenting with a lump and altered sensation in the breast: a case report

**DOI:** 10.1186/1752-1947-3-6442

**Published:** 2009-03-10

**Authors:** Simon T Adams, Julie Cox, G Sam Rao

**Affiliations:** 1 University Hospital of North Durham, North Road, Durham, County Durham DH1 5TW, UK

## Abstract

**Introduction:**

Silicone lymphadenopathy is a rare but recognised complication of procedures involving the use of silicone. It has a poorly understood mechanism but is thought to occur following the transportation of silicone particles from silicone-containing prostheses to lymph nodes by macrophages.

**Case presentation:**

We report of a case involving a 35-year-old woman who presented to the breast clinic with a breast lump and altered sensation below her left nipple 5 years after bilateral cosmetic breast augmentations. A small lump was detected inferior to the nipple but clinical examination and initial ultrasound investigation showed both implants to be intact. However, mammography and magnetic resonance imaging of both breasts revealed both intracapsular and extracapsular rupture of the left breast prosthesis. The patient went on to develop a flu-like illness and tender lumps in the left axilla and right mastoid regions. An excision biopsy of the left axillary lesion and replacement of the ruptured implant was performed. Subsequent histological analysis showed that the axillary lump was a lymph node containing large amounts of silicone.

**Conclusion:**

The exclusion of malignancy remains the priority when dealing with lumps in the breast or axilla. Silicone lymphadenopathy should however be considered as a differential diagnosis in patients in whom silicone prostheses are present.

## Introduction

Silicone has been used in surgery for over 30 years in procedures such as joint replacement and breast augmentation. Initially, it enjoyed a reputation as being a biologically inert substance. Over the past 15 to 20 years, however, concerns over the safety of silicone implants have culminated in several well-publicised legal cases and negative media reports. Its use has been curtailed for fears of association with granulomatous reactions and, in rarer cases, malignancy [1,2]. Injections of free silicone into breast tissue have long been abandoned in the United States following the development in some women of disfiguring complications such as gravitational migration through the soft tissues to distant sites [1,3].

Silicone is a non-biodegradable prosthetic material which elicits relatively little local inflammation in most people due to its low tissue immunogenicity [3]-[6]. It is composed of dimethylsiloxane polymers which can result in differing properties and products according to the variation in their chain lengths and cross-links [4,7]. Liquid silicone consists of short chains, and gels are made from long chains [4].

Despite its initial reputation as a biologically inert substance, it has been associated in the literature with numerous, albeit rare, complications including local and systemic granulomatous inflammatory reactions affecting breast tissue, lymph nodes, joint capsules, the heart, liver and kidneys. In addition, it has been suggested that silicone may be a causative factor in the development of adult respiratory distress syndrome (ARDS), various connective tissue and autoimmune diseases and human adjuvant disease [4,8,9]. At present, the mechanism of such complications is uncertain and in some cases, proof of such a relationship remains a source of controversy [2,9]-[11]. Malignant lesions including lymphoma and cancers of the breast and lung have arisen in those with silicone prostheses although again there has yet to be any firm proof of its carcinogenicity. Indeed, some papers have shown a reduced relative risk of breast cancer in women with breast implants [1,4,12]. The inflammatory reaction is thought to be more pronounced in the lymph nodes than in connective tissue [1,8].

Silicone particles can migrate through tissues following either rupture or erosion of a silicone-containing surface or through continued leakage through an intact surface [1,3,4,6,8]. The risk of rupture and/or leakage increases with increasing age of the implant, the site of implantation (retroglandular as opposed to submuscular), the presence of local tissue contractures and/or symptoms and the type and/or manufacturer of the implant used [6,7,13]. The average age at rupture varies between studies but is in the region of 10 to 13 years and it is best diagnosed with magnetic resonance imaging (MRI) scanning [4,14]. Rupture itself is normally a relatively harmless condition which only rarely progresses and becomes symptomatic [15].

When leakage does occur, silicone can cause fibrosis and foreign body granulomatous reactions, especially when combined with certain fatty acids, resulting in pain and contractures [4,6]. Once silicone particles have breached the confines of their prosthesis and passed through any local fibrotic reaction, they may be transported to regional lymph nodes by macrophages in the reticulo-endothelial system [1]. The resulting granulomatous reactions may present as lymphadenopathy and, when sited in the axilla, malignancy of the ipsilateral breast is a diagnosis which needs to be excluded. Indeed, it is not impossible for both silicone granulomata and breast cancer metastases to coexist in the same lymph node [6].

Silicone lymphadenopathy has been reported more frequently following joint surgery than following breast augmentation either by silicone gel implants or silicone injection [1,6]. When associated with breast augmentation, it primarily affects the axillary nodes but cases have been reported involving intramammary, internal mammary and supraclavicular nodes [3,5].

Fine needle aspiration (FNA) of affected lymph nodes has been shown to be a cost effective and accurate method of excluding malignancy and diagnosing implant disruption in patients with silicone prostheses presenting with an axillary mass [6]. Under such circumstances, fine needle aspiration cytology (FNAC) shows a foreign body reactive lymphoid background with numerous giant cells [1,6]. Specifically, one sees cystic spaces with multivacuolated macrophages but relatively few multinucleated giant cells [1,8]. Other granulomatous processes can be excluded if birefringent particles are found in the macrophages whereas in silicone reactive macrophages, the vacuoles contain refractile, homogenous and faintly yellow non-birefringent material [1,3,6].

Some papers have suggested that a conservative approach involving excision of the axillary nodes is favourable. The rationale for this is that silicone granulomata have been found as incidental findings in axillary nodes removed at mastectomy for breast cancer in the presence of intact breast prostheses [1]. Also it has been suggested that silicone may dilute the cellular elements within the node and thus mask the presence of cancer cells [1]. If intramammary nodes are affected, then excision has been recommended as mammography is unable to differentiate between benign and malignant pathology [5].

## Case presentation

A 35-year-old British Caucasian woman was referred by her general practitioner to our breast symptomatic services following a 3-week history of a lump below her left nipple. She also complained of some itchiness and a hot feeling in the same region. She had undergone bilateral breast augmentation using subglandular cohesive gel silicone implants 5 years previously.

Clinical examination revealed soft healthy implants which were clinically intact. There was a 2 mm mobile lump behind the left nipple.

Initial ultrasound investigation showed both implants to be intact but there were multiple hypoechoic areas at the symptomatic site in the left retroareolar region which appeared superficial to the implant. Mammography showed an irregular contour of the left implant. A magnetic resonance imaging (MRI) scan of both breasts was suggestive of both intracapsular and extracapsular rupture of the left breast prosthesis.

**Figure 1:**
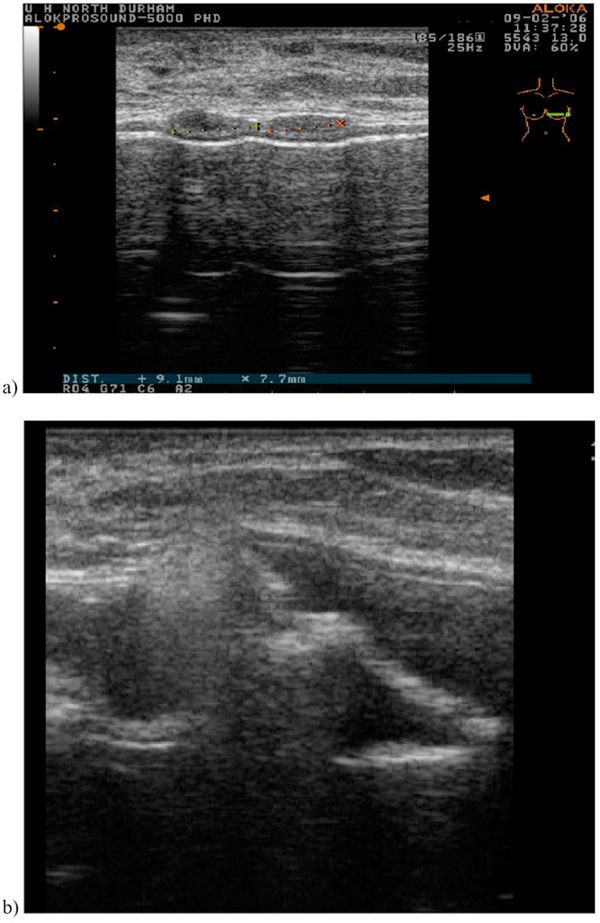
**Ultrasound of the left breast implant**. Demonstrates several hypoechoic areas measuring 9 and 7mm, respectively, suggestive of extracapsular rupture **(a)** with gross disorganisation of the internal implant structure in keeping with intracapsular rupture **(b)**.

**Figure 2:**
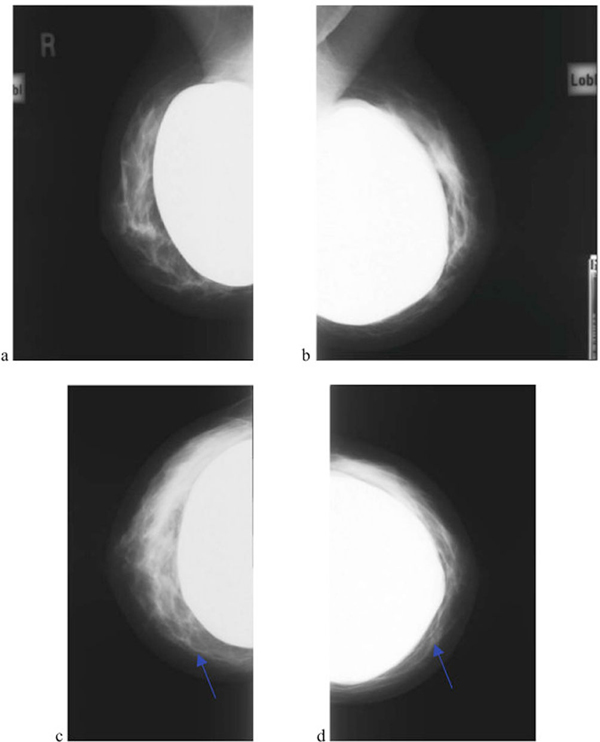
**Bilateral mammography **(a)**–**(d)****. Demonstrates irregularity of the contour of the left breast implant (blue arrow).

**Figure 3:**
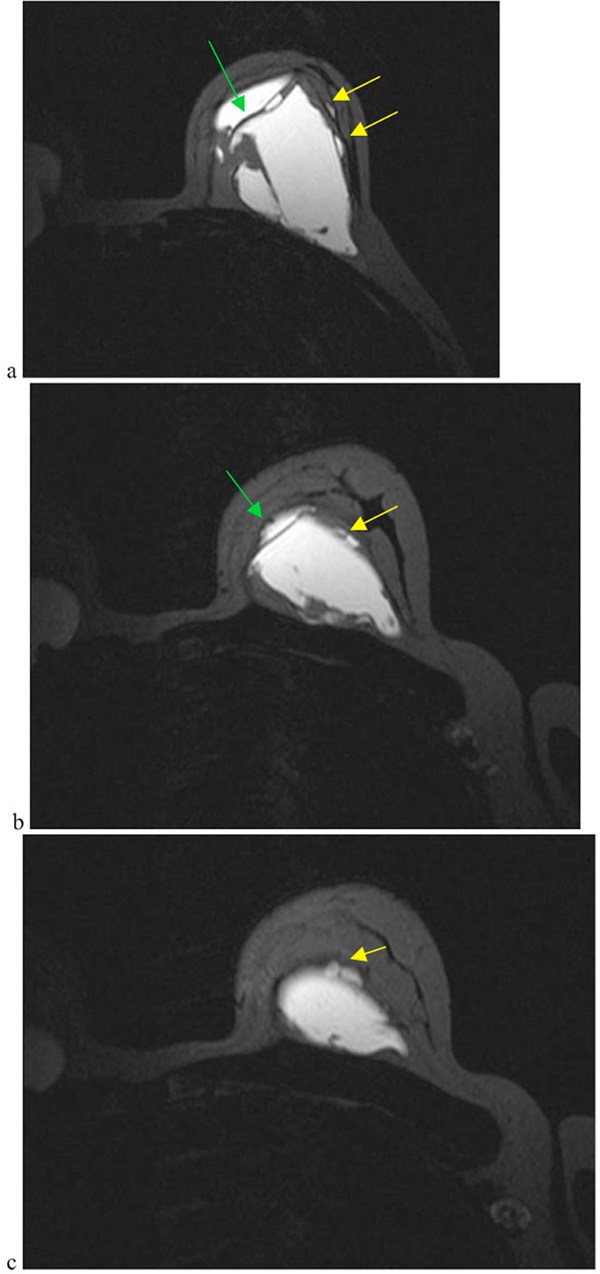
**Magnetic resonance imaging of the breasts**. Axial T1 weighted fat suppressed images through the left implant **(a)–(c)** demonstrate extracapsular silicone (yellow arrow) with gross disorganisation and collapse of the implant with a positive "linguine sign" (green arrow). Features are of a collapsed intra and extracapsular rupture.

The patient was seen in the breast clinic with the results of the radiological investigations (Figures 1, 2 and 3). On this occasion, she complained of a tender lump in the left axilla after a flu-like illness. Clinically, the lump was thought to be a lymph node and following review a month later, she was listed for excision biopsy of the axillary lesion.

Before the excision biopsy, the patient was reviewed as an outpatient by the plastic surgeon who had performed the original augmentation procedure. At this time, a similar tender lump to the left axillary mass was found in the right mastoid region.

**Figure 4:**
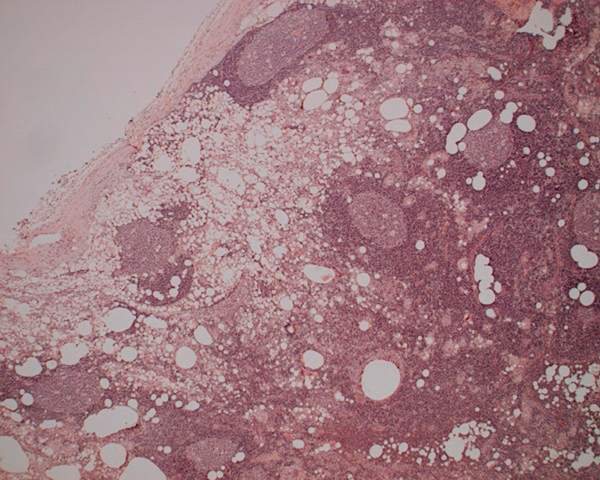
**Lymph node (×40)**. Demonstrates the subcapsular sinus diffusely expanded by vacuolated histiocytes.

**Figure 5:**
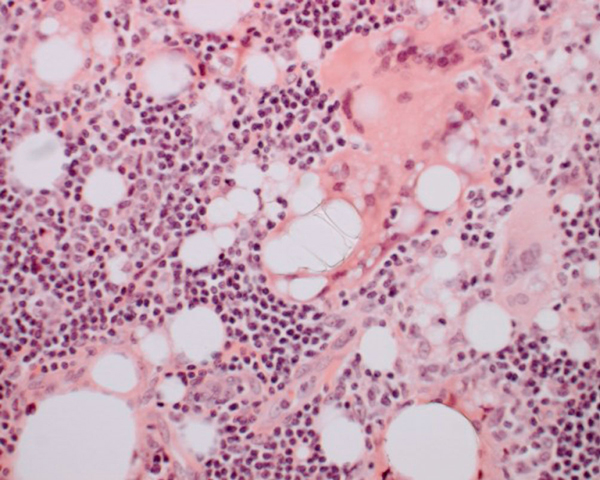
**Multinucleate giant cells (×200)**. Demonstrate vacuoles some of which contain refractile material consistent with silicone.

A combined procedure involving excision biopsy of the left axillary lesion and replacement of the ruptured implant was performed. Pus-like fluid was seen to surround the ruptured implant and ooze from the axillary node. Subsequent histological analysis showed that the axillary lymph node contained large amounts of silicone and demonstrated a lipogranulomatous reaction (Figures 4, 5).

Two weeks postoperatively, the patient had clinically improved with resolution of her operative discomfort.

## Discussion and Conclusion

This case demonstrates the need to retain an open mind when dealing with lumps in the breast and axilla and also reinforces the need to employ a high index of suspicion. Silicone lymphadenopathy is a rare complication of procedures involving insertion of silicone-containing prostheses and, whilst the diagnosis must be considered, the need to exclude malignancy histologically is paramount.

## Abbreviations

ARDS: adult respiratory distress syndrome; FNA: fine needle aspiration; FNAC: fine needle aspiration cytology; MRI: magnetic resonance imaging.

## Consent

Written informed consent was obtained from the patient for publication of this case report and any accompanying images. A copy of the written consent is available for review by the Editor-in-Chief of this journal.

## Competing interests

The authors declare that they have no competing interests.

## Authors' contributions

STA was the primary author of the manuscript; JC provided critical appraisal and re-writing of initial drafts of the manuscript, and provided the radiological imaging and legends. SR was the senior author, and provided critical appraisal and re-writing of drafts of the manuscript.
